# Onco-testicular sperm extraction (Onco-TESE) from a single testis with metachronous bilateral testicular cancer: a case report

**DOI:** 10.1186/s12610-018-0066-2

**Published:** 2018-01-25

**Authors:** Itsuto Hamano, Shingo Hatakeyama, Rika Nakamura, Rie Fukuhara, Daisuke Noro, Hiroko Seino, Takahiro Yoneyama, Yasuhiro Hashimoto, Takuya Koie, Yoshihito Yokoyama, Chikara Ohyama

**Affiliations:** 1 Department of Urology, Hirosaki University Graduate School of Medicine, 5 Zaifu-chou, Hirosaki, 036-8562 Japan; 2 Department of Obstetrics and Gynecology, Hirosaki University Graduate School of Medicine, Hirosaki, Japan; 3 Department of Pathology and Bioscience, Hirosaki University Graduate School of Medicine, Hirosaki, Japan; 4 Department of Advanced Transplant and Regenerative Medicine, Hirosaki University Graduate School of Medicine, Hirosaki, Japan

**Keywords:** Onco-TESE, Testicular cancer, Azoospermia, Intracytoplasmic sperm injection (ICSI), Onco-TESE, Cancer testiculaire, Azoospermie, Injection intra-cytoplasmique d’un Spermatozoïde (ICSI)

## Abstract

**Background:**

Although oncologic testicular sperm extraction (onco-TESE) has been increasingly practiced, the evidence of onco-TESE performed in patients with testicular cancer is insufficient. Furthermore, in bilateral testicular cancer, accounting for 0.5%–1% of testicular cancers, onco-TESE is more challenging and has been insufficiently reported.

**Case presentation:**

Here we report the case of a 25-year-old man who underwent onco-TESE from his residual single testis with a nonseminomatous germ cell tumor that occurred 5 years after orchiectomy of the contralateral testis. A second orchiectomy and simultaneous TESE from the noncancerous testicular tissue were performed. The pathological diagnosis was germ cell tumors, tumors of more than one histological type (embryonal carcinoma, immature teratoma, yolk sac tumor, seminoma, and choriocarcinoma; pT1N0M0). The patient subsequently married and hoped for fatherhood 3 years later. Whereas histological diagnosis of the normal testicular tissue was Johnsen score 6 (maturation arrest), morphologically normal and motile sperms were successfully retrieved from thawed TESE samples and used for multiple cycles of intracytoplasmic sperm injection. Although the conception has not been succeeded to date, ICSI attempts have been continuing.

**Conclusion:**

This case demonstrates the effectiveness of onco-TESE for challenging cases such as bilateral and nonseminmatous testicular cancer.

## Résumé

**Contexte:**

Bien que l’extraction oncologique de spermatozoïdes testiculaires (onco-TESE) soit une pratique croissante, le bien-fondé de réaliser une onco-TESE chez des patients qui ont un cancer du testicule reste insuffisamment étayé. Par ailleurs, en cas de cancer bilatéral, qui représente 0,5–1% des cancers du testicule, l’onco-TESE est. plus difficile, et peu de cas ont été rapportés.

**Cas clinique:**

Nous rapportons le cas d’un homme de 25 ans qui a bénéficié d’une onco-TESE pour tumeur à cellules germinales non séminomateuse sur testicule unique résiduel, survenant 5 ans après une orchidectomie controlatérale. Ont été réalisées simultanément une seconde orchidectomie et une TESE sur tissu testiculaire non tumoral. L’étude anatomopathologique a montré des tumeurs à cellules germinales de plus d’un type histologique (carcinome embryonnaire, tératome immature, tumeur vitelline, séminome, et choriocarcinome; pT1N0M0). Le patient a ensuite convolé en noces et le couple a souhaité avoir un enfant 3 ans plus tard. Alors que l’étude histologique du tissu testiculaire normal donnait un score de Johnsen à 6 (arrêt de maturation), des spermatozoïdes morphologiquement normaux et mobiles ont été retrouvés dans les échantillons de TESE décongelés; ces spermatozoïdes ont été utilisés pour réaliser plusieurs cycles d’injection intra cytoplasmique. Bien qu’aucune conception n’ait eu lieu à ce jour, les tentatives d’ICSI se poursuivent.

**Conclusions:**

Ce cas montre l’efficacité de l’onco-TESE face à des cas tels qu’un cancer testiculaire bilatéral et non séminomateux.

## Background

Although oncologic testicular sperm extraction (onco-TESE) has been increasingly practiced, the evidence of onco-TESE performed in patients with testicular cancer, especially bilateral testicular cancer, is insufficient. We present a challenging case of onco-TESE from a single cancerous testis with a metachronous bilateral testicular tumor that was detected at 5 years after orchiectomy of the contralateral testis; this procedure resulted in the successful retrieval of normal sperms, which were used for intracytoplasmic sperm injection (ICSI).

## Case presentation

**Fig. 1:**
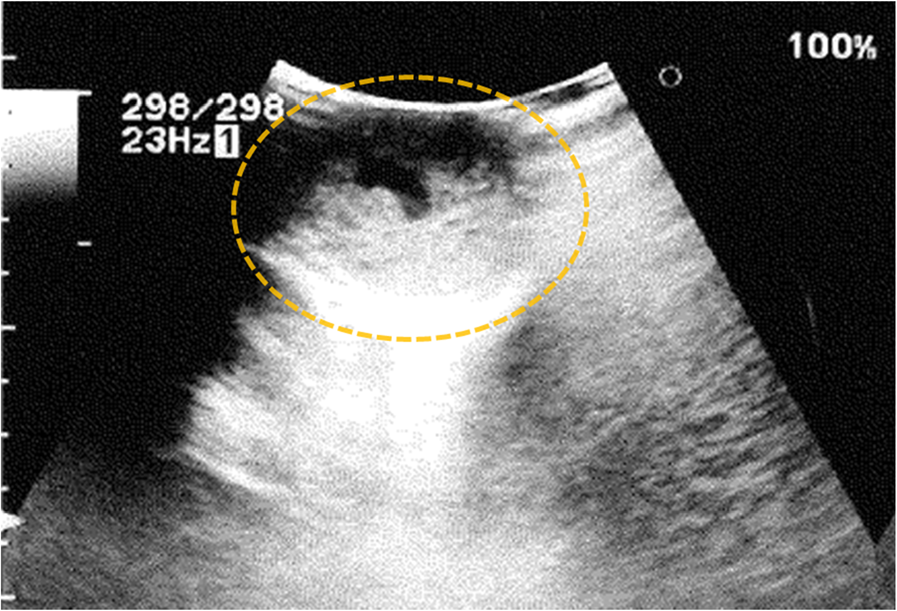
Ultrasonographic findings in the patient. Dotted circle indicates a mass with a heteroechoic pattern and 4-cm diameter

**Fig. 2:**
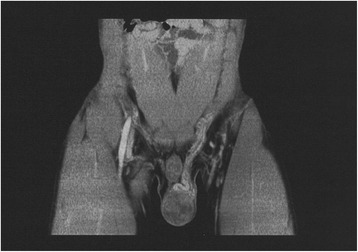
Computed tomography findings in the patient. A partially enhanced localized tumor was detected in his left testis, with no distant metastasis or lymph node swelling

**Fig. 3:**
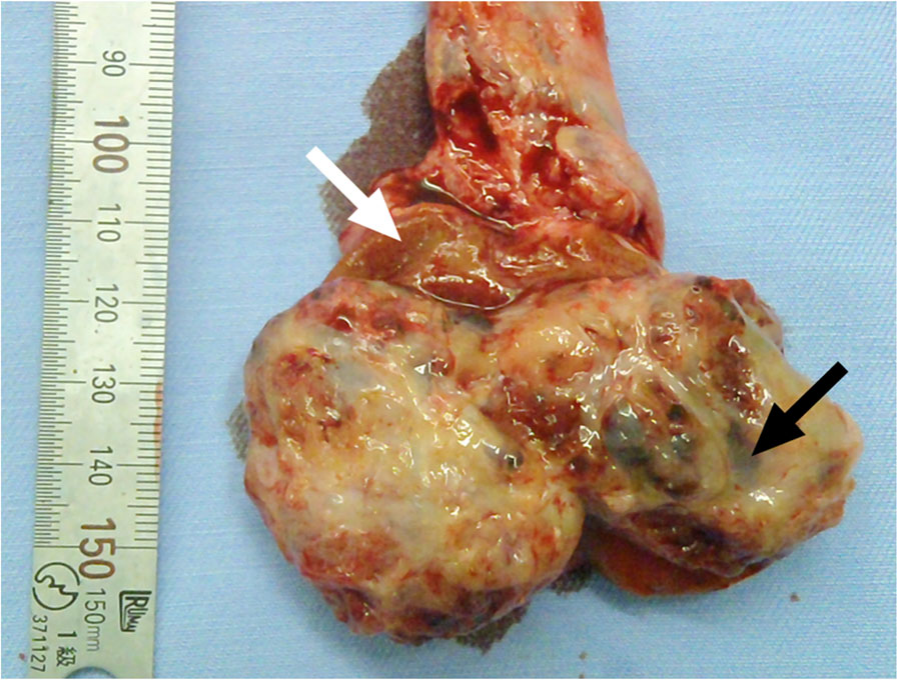
Macroscopic appearance of the resected testis. The white arrow indicates the macroscopically recognized normal testicular tissue that was subsequently cryopreserved. The black arrow indicates the testicular tumor

**Fig. 4:**
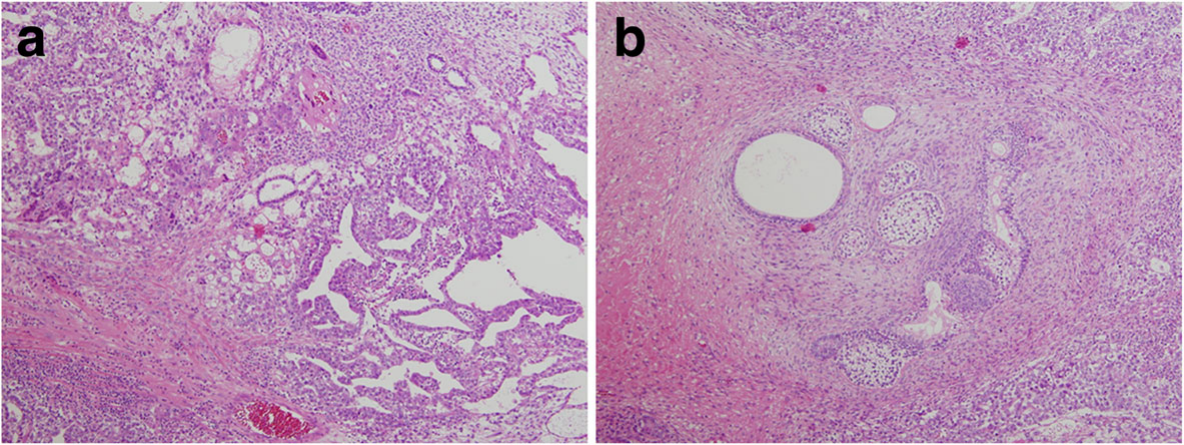
Histopathological findings of the resected testicular tumor. Hematoxylin–eosin stain of the embryonal carcinoma and yolk sac tumor (100×) (**a**) and immature teratoma (100×) (**b**)

**Fig. 5:**
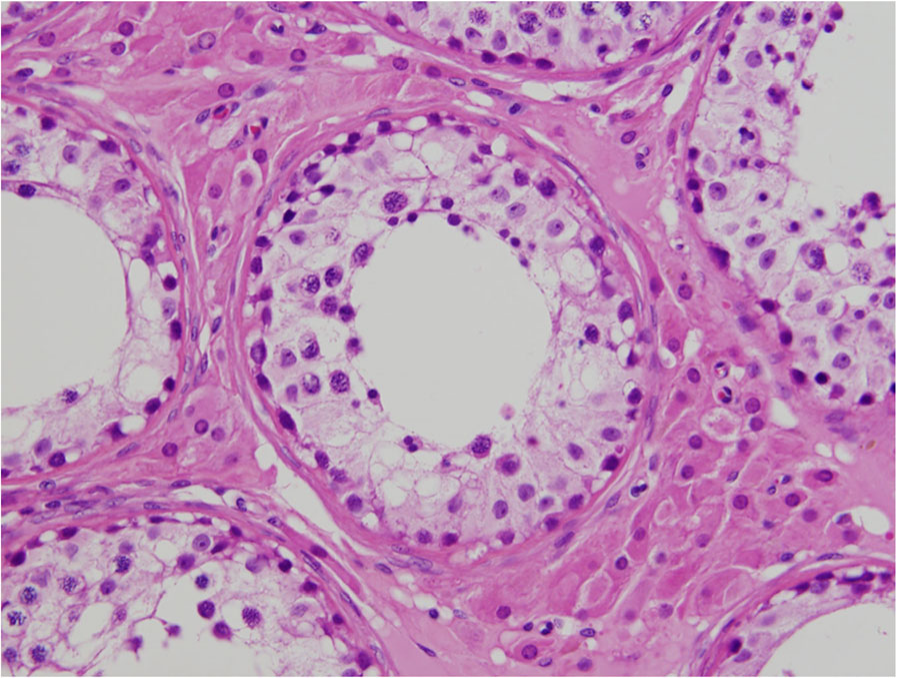
Histopathological findings of resected normal testicular tissue. Numerous seminiferous tubules, including Sertoli cells, spermatogonia, spermatocytes, and several early round spermatids but no late spermatid were identified, indicating late maturation arrest (Johnsen score, 6) (400×)

A 25-year-old unmarried Japanese man presented to our hospital with a mass in his left testis. He underwent orchiectomy of his right testis 5 years earlier, and histopathological diagnosis revealed immature teratoma of the testis. No salvage chemotherapy was performed. He discontinued follow up several years after the surgery on account of moving away. Five years after the orchiectomy, his residual left testis was noted to be slightly enlarged, and a nodule was palpable. The volume of normal testicular was not measurable due to replacement by the tumor. Ultrasonography revealed a mass with a heteroechoic pattern and 4-cm diameter (Fig. 1). Laboratory blood tests revealed elevated alfa-fetoprotein (AFP, 484 ng/mL) and human chorionic gonadotropin (hCG, 643.4 mIU/mL) levels. Computed tomography revealed a partially enhanced localized tumor in his left testis, without any distant metastasis or lymph node swelling (Fig. 2). We diagnosed a testicular tumor in his residual left testis without distant metastasis, and a second orchiectomy was planned. Semen analyses were performed twice prior to surgery, and both test results indicated unobstructed azoospermia. No physical findings or complaints regarding varicocele were recognized. Because of the patient’s desire for future fertility, we opted for simultaneous orchiectomy and onco-TESE from the normal tissue of the resected testis. Immediately after the removal of the testis, we sampled the normal testicular tissue that was macroscopically divided from cancer tissue (Fig. 3). Rapid microscopic observation of the normal testicular tissue revealed a few motile sperms, and the tissue was immediately cryopreserved after separation into 41 samples. The pathological diagnosis was a germ cell tumor comprising tumors of more than one histological type (pT1N0M0; embryonal carcinoma 50%, immature teratoma 30%, yolk sac tumor 10%, seminoma 5%, and choriocarcinoma 5%; Fig. 4). The macroscopically normal testicular tissue obtained through TESE contained numerous seminiferous tubules, including Sertoli cells, spermatogonia, spermatocytes, and several early round spermatids but no late spermatid. The histological diagnosis was late maturation arrest (Johnsen score, 6; Fig. 5). His serum testosterone, AFP, and hCG levels decreased to normal levels after surgery, and testosterone enathate (250 mg) was intramuscularly injected once per month. He received two cycles of BEP (bleomycin, etoposide, and cisplatin) and has had no recurrence to date. Three years after surgery, he was married and opted for ICSI using his preserved sperms. The cryopreserved testicular tissue was partially thawed at the time of use for multiple cycles of ICSI. In 6 of 14 ICSI cycles, morphologically normal and motile sperms were injected, and fertilized eggs developed into blastocysts. To date, conception has not yet been achieved. However, the 27 samples of testicular tissue remain, and the couple is willing to continue attempting conception with ICSI.

## Discussion

Although testicular cancer is one of the most common malignancies in men of reproductive age [1], the treatment modalities are often detrimental to male fertility [2]. Therefore, fertility preservation is often a critical issue among young male patients with testicular cancer. Currently, sperm cryopreservation before gonadotoxic cancer treatment is only the established procedure for fertility preservation. For patients with nonobstructive azoospermia, TESE is a commonly selected method to directly retrieve seminiferous tubules from testicular tissue. Considering the emphasis on sustaining the quality of life of cancer survivors in the last few decades, onco-TESE has been increasingly practiced since its introduction by Schrader in 2003 [3]. Testicular cancer patients more frequently need onco-TESE due to the higher risk of azoospermia. The incidence rate of azoospermia before any sterilization treatment is 5–10% among testicular cancer patients, which is significantly higher than the 1% in the normal population [4–6].

The methodology for onco-TESE in testicular cancer patients has not been established. Onco-TESE for testicular cancer patients is often challenging because the sample needs to be retrieved from noncancerous tissue that has been separated from cancerous tissue. The evidence of using sperms from noncancerous tissue in cancerous testes has not been sufficiently accumulated. To our knowledge, only 27 cases of onco-TESE in testicular cancer patients have been reported in the literature [3, 6–11]. This case report showed the result of onco-TESE with bilateral testicular cancer, which is more challenging than one with unilateral cancer. Most testicular cancers occur unilaterally, and the incidence rate of bilateral cancer is only 0.5–5% [12]. Among bilateral testicular cancer cases, approximately 35% are identified as synchronous and 65% are metachronous [13]. Our case is a metachronous type occurring in his residual testis 5 years after the first orchiectomy in the contralateral testis. In terms of occurrence patterns, onco-TESE in testicular cancer can be categorized into three types: (i) TESE from the contralateral normal testis at the time of orchiectomy for a unilateral testicular tumor, (ii) TESE from the healthier side of a testis with a synchronous bilateral testicular tumor, and (iii) TESE from a single testis with a metachronous testicular tumor in patient who formerly underwent orchiectomy. The 27 reported cases of onco-TESE in testicular cancer included 20 cases of type (i), 4 cases of type (ii), and 3 cases of type (iii). Our case presents the fourth experience of type (iii) [3, 6–11].

It is noteworthy that the normal sperms were successfully harvested and used for multiple ICSI cycles in this case, because the outcomes of onco-TESE, including the success rates of sperm retrieval, use, conception, and pregnancy, have not been sufficiently documented. Among the reported cases of onco-TESE, the success rate of sperm retrieval was 45% in the azoospermia patents with any type of cancer and 59% (16 of 27 cases) in those with testicular cancer [3, 6–11]. These rates do not seem significantly lower than the successful retrieval rates of 30–60% for TESE in non-cancer patients [14]. The usage/pregnancy rates after onco-TESE remain even more unclear because of the limited number of cases and short period of observation. Among 16 reported cases of successful sperm retrieval with onco-TESE in testicular cancer patients, only 5 cases underwent ICSI, and 4 cases resulted in pregnancy [7–10]. Our case is the third documented case of bilateral metachronous testicular cancer resulting in sperm use for ICSI, and the first case of a nonseminomatous testicular cancer with such an occurrence pattern and actual use of preserved sperms [8, 9].

The safety of using sperms retrieved from cancerous testis in terms of fetal health also remains unclear. Only a few cases have reported birth of healthy baby with onco-TESE from cancerous testis, including one case with bilateral testicular cancer [7–10]. Although a previous study has reported that smaller tumor diameter and longer distance from tumor margin were positive predictors of favorable spermatogenesis with onco-TESE in testicular cancer patients [15], it is indeterminate whether onco-TESE with such favorable spermatogenesis is secure. Accumulative evidences for the safety of onco-TESE are necessary.

The present case also suggested the possibility of sperm retrieval with onco-TESE despite unfavorable Johnsen score. Although the Johnsen score in this case was 6, indicating maturation arrest, normal sperms were obtained from the cryo-thawed testicular tissue. This discrepancy occurs possibly because the onco-TESE was performed with the macroscopic detection of normal testicular tissue. Conversely, microdissection TESE (MD-TESE) that enables sampling from microscopically healthier seminiferous tubules may be recommended for onco-TESE. Additionally, this case also showed an inconsistent result between histological and clinical information: the wide intraseminiferous lumen suggesting obstruction of the tubules in contrast to the clinical diagnosis of non-obstructive azoospermia with the elevated sexual hormones. Although it is difficult to clarify the reason for this inconsistency, this case may imply that such dilated intraseminiferous lumen is caused by partial obstruction of the tubules in the microenvironment of cancerous testis in azoospermic patients with testicular cancer.

## Conclusion

In conclusion, our case presented that motile normal sperms were successfully retrieved from preserved testicular tissue and used for multiple ICSI attempts. Although successful pregnancy has not yet been achieved in this case, the successful experience of sperm retrieval from a residual single testis in a patient with bilateral and metachronous nonseminoma implies the effectiveness of onco-TESE in challenging cases.

## Abbreviations

AFP: Alfa-fetoprotein
hCG: Human chorionic gonadotropin
ICSI: Intracytoplasmic sperm injection
MD-TESE: microdissection TESE
Onco-TESE: Oncologic testicular sperm extraction
TESE: Testicular sperm extraction
